# Chinese newspaper coverage of genetically modified organisms

**DOI:** 10.1186/1471-2458-12-326

**Published:** 2012-06-08

**Authors:** Li Du, Christen Rachul

**Affiliations:** 1 Health Law and Science Policy Group, Faculty of Law, University of Alberta, T6G 2H5, Edmonton, AB, Canada

## Abstract

**Background:**

Debates persist around the world over the development and use of genetically modified organisms (GMO). News media has been shown to both reflect and influence public perceptions of health and science related debates, as well as policy development. To better understand the news coverage of GMOs in China, we analyzed the content of articles in two Chinese newspapers that relate to the development and promotion of genetically modified technologies and GMOs.

**Methods:**

Searching in the Chinese National Knowledge Infrastructure Core Newspaper Database (CNKI-CND), we collected 77 articles, including news reports, comments and notes, published between January 2002 and August 2011 in two of the major Chinese newspapers: *People’s Daily* and *Guangming Daily*. We examined articles for perspectives that were discussed and/or mentioned regarding GMOs, the risks and benefits of GMOs, and the tone of news articles.

**Results:**

The newspaper articles reported on 29 different kinds of GMOs. Compared with the possible risks, the benefits of GMOs were much more frequently discussed in the articles. 48.1% of articles were largely supportive of the GM technology research and development programs and the adoption of GM cottons, while 51.9% of articles were neutral on the subject of GMOs. Risks associated with GMOs were mentioned in the newspaper articles, but none of the articles expressed negative tones in regards to GMOs.

**Conclusion:**

This study demonstrates that the Chinese print media is largely supportive of GMOs. It also indicates that the print media describes the Chinese government as actively pursuing national GMO research and development programs and the promotion of GM cotton usage. So far, discussion of the risks associated with GMOs is minimal in the news reports. The media, scientists, and the government should work together to ensure that science communication is accurate and balanced.

## Background

Genetically modified organism (GMO), refers to an organism whose genetic material has been altered by genetic engineering techniques. With these advancements in agricultural biotechnology, desired products can be realized in both genetically modified (GM) plants and animals. For instance, GM biotechnology can increase quantities of food production, lower pesticide use [1] and yield products with particular traits, e.g., “Golden Mustard”, which can express high levels of beta-carotene, a precursor of Vitamin A, for treating Vitamin A deficiency [2]. Based on the advantages of GMOs, big seed companies in developed countries, mainly in the United States (US), launched research and development (R&D) programs for GM technology in the early 1980s [3]. With the help of the National High-Tech R&D Program (commonly known as National 863 Program), National Basic Research Program of China (973 Program) [4] and special programs for transgenic technology research, China has made significant progress on GM cotton technology since 1996 [5]. Because of these developments, the Chinese cotton industry has increased the yield and quality of cotton, and has successfully broken the monopoly of international companies [6]. According to Reuters, China has become the largest producer of GM cotton in the world [7].

In addition to the progress in GM technologies, China has also developed a regulatory framework that places great importance on the bio-safety management of agricultural GMOs. The State Council promulgated the Regulation on Safety Administration of Agricultural GMOs on May 23, 2001, and later, on January 5, 2002, the Ministry of Agriculture issued three supporting regulations to facilitate the completion of bio-safety regulation on GMOs [8]. In 2004, the State Administration of Quality Supervision, Inspection and Quarantine issued Administrative Measures for Entry/Exit Inspection and Quarantine of GM Products [9]. China has also initially established a bio-safety management system for agricultural GMOs and a National Agricultural GMOs Bio-safety Committee. The bio-safety management system is composed by an Inter-ministerial Joint Conference System for Bio-safety Management, which is composed by 7 concerned national ministries. With regard to the National Agricultural GMO Bio-safety Committee, its main responsibility is to provide technical supports in GMO bio-safety management [10].

Despite the growing use and popularity of GMOs, scientists have not yet fully explored the potential environmental and health risks of GMOs. Some scientists and social organizations are worried that GMOs may create potential health and environmental risks, such as food allergy [11], genetic erosion, and increased vulnerability of crop plants to pests and diseases [12]. In addition to potential environmental and health risks, there are a number of social and ethical issues also associated with GM technology, including the commodification of life and an increase in inequality [13]. It is based on these issues that GMOs, especially GM food, remains controversial.

In China, the focus of public attention is mainly on the health issues associated with GM food. Although the commercial production of GM rice and corn are not approved in China, the safety approval for two kinds of GM strains of rice and one type of corn, given by the Ministry of Agriculture’s bio-safety committee in November 2009 [14], has led to great debate in the country about whether genetically modified rice, the staple food for the majority of Chinese, is safe for consumption. However, according to the article *Breaking Chinese-style Fallacies and Rumors about GMOs*, originally published on Nanfang Zhoumo and later cited by Ministry of Agriculture on its website, most of the doubts about the safety of GM food are fallacious. It claimed that those rumors were due to the public’s ignorance about biotechnology and the loss of confidence in government authority [14].

The news media can play an important role in informing the public about new technologies, such as GMOs [15]. The news media can both reflect and shape public perceptions about new health and science developments, and some have suggested that media can have an influence on policy development [16]. While the extent of the news media’s influence, both on public perceptions and on policy development, may differ across jurisdictions, it has been observed that the news media in China helps to popularize scientific knowledge and aims to mitigate the public’s doubts about new inventions [15]. Key differences in the role of media in China, as compared to Western nations, may also reflect the degree to which Chinese media may reflect government agendas [17]. Moreover, although traditional media are no longer the only source of information (i.e., Internet) [18], newspapers are still an important source of health information that can help to frame issues, as well as perceptions of the risks and benefits of new technology [19].

Despite the important role that media plays, little has been written about how the Chinese media have portrayed GMOs. We conducted an analysis of Chinese leading national media coverage of GMOs in order to understand whether GMOs are portrayed positively or negatively, and how much and what kind of information is provided about GMOs. The analysis included print news reports, from the *People’s Daily* and *Guangming Daily*, with the objective of reviewing the content of media coverage, including what kinds of GMOs have been mentioned; which issues are associated with GMOs; mention of benefits and risks associated with GMOs; and theme, i.e., whether the article supports, opposes or is neutral about research, development, and adoption of GM technology.

## Methods

To develop a sample of newspaper articles that included discussion of GMOs, we collected articles from the newspapers *People’s Daily* and *Guangming Daily*. These two newspapers are major Communist Party newspapers and considered the ‘elite’ press in China [20,21], and as such have a role in agenda setting [22]. However, since they serve as official newspapers in China, they may not be representative of all Chinese print newspapers. We searched the Chinese National Knowledge Infrastructure Core Newspaper Database (CNKI-CND), an academic literature full-text database which covers the full content of the *People’s Daily* and *Guangming Daily* since 2000.

Using the search term “” (“genetically modified”), between the dates of January 1, 2002 to August 31, 2011, we collected 50 articles published in the *People’s Daily* and 27 articles in the *Guangming Daily* that have “GMO” in the titles and are relevant to GMOs. We chose to search titles only, and not the entire article, to ensure that news articles included extensive discussion of GMOs.

Articles were analyzed using an inductive method to gather information about types of GMOs discussed in news articles, issues associated with GMOs, and benefits and risks associated with GMOs. The attitude of news articles towards GMOs, which was whether the articles were supporting, opposing or neutral in regards to GMOs, was also assessed. This attitude includes both the author’s attitude toward GMOs and/or the “sides” presented in the news article. For example, an author may be writing in a neutral tone, but only opposing opinions are presented - this article would be classified as questioning/opposing. If the title of an article was negative, but the author wrote in a neutral tone; this article was classified as neutral.

## Results

### Types of GMOs

The news articles covered both GM plants and animals. Specifically, 29 kinds of GMOs have been reported by the Chinese media (see Table 1). Notably, 37.7% of newspaper articles were dedicated to GM cotton, covering a variety of issues from the advantages of cultivation of GM cotton to the new progress in GM cotton technology research in China. Other frequently mentioned GM plants were GM corn (30.8%), soybean (29.9%), rice (24.7%), and Canola (18.2%). For GM animals, 5.2% of all articles reported on GM sheep. GM pig, rabbit and cow were mentioned in 3.9% of newspaper articles respectively.

**Table 1:** Types of GMOs mentioned in news articles

**Type of GMO**	**% (#) of Articles**
**GM Plants**	
Cotton	37.7% (29)
Corn	30.8% (24)
Soybean	29.9% (23)
Rice	24.7% (19)
Canola	18.2% (14)
Wheat	7.8% (6)
Potato	6.5% (5)
Tomato	6.5% (5)
Others	≤3.9% (3)
**GM Animals**	
Sheep	5.2% (4)
Pig	3.9% (3)
Rabbit	3.9% (3)
Cow	3.9% (3)
Others	1.3% (1)

### Issues concerning GMOs

**Figure 1:**
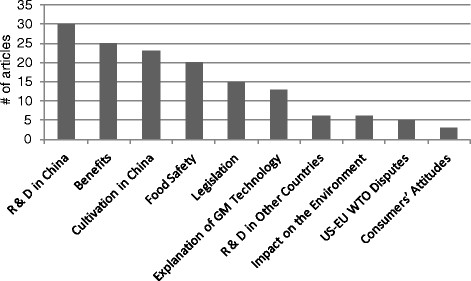
Issues relating to GMOs in newspaper articles.

The Chinese media articles in our data set included discussion of 10 different issues (see Figure 1). Among these issues, the most frequently discussed by the Chinese media were: the development of GM technology in China (e.g., breakthroughs in Chinese GM cotton research [23]); the benefits of GMOs (e.g., GM technology can help to increase crop yields while reducing pesticide use [24]); the cultivation of GMOs in China (e.g., from 2008 to 2010, the cultivation of GM cotton in china was extended up to 28 million acres [25]); and safety issues associated with GM food (e.g., whether or not Bt Protein is safe for eating [26]).

### Pros and cons of GMOs

The news articles were also assessed for discussion of benefits and risks of the development and adoption of GM technology. During analysis, 9 benefits of GMOs were identified and 7 potential risks were identified (see Table 2). The two most common benefits of GMOs were the decreased use of pesticides or herbicides, and higher yields, each accounting for 33.8% of all articles (26 articles). Other frequently mentioned advantages include: lowering cost (29.9%), improving quality (27.3%) and conferring tolerance to drought, saline soil or cold (22.1%). Four articles stated the environmental benefits of GMOs. Three of them reported that GMOs can decrease phosphorus emissions, and one article indicated that GMOs can reduce greenhouse gas emissions.

**Table 2:** Benefits and risks of GMOs mentioned in newspaper articles

**Benefits of GMOs**	**% (#) of Articles**
Less pesticide or herbicide use	33.8% (26)
Higher yields	33.8% (26)
Lower cost	29.9% (23)
Quality improvement	27.3% (21)
Tolerance to drought, saline soil or cold	22.1% (17)
Food security	14.3% (11)
Decrease phosphorus emissions	3.9% (3)
Decrease greenhouse gas emissions	1.3% (1)
Restrain the spread of disease	1.3% (1)
**Risks of GMOs**	
Human health safety problems	11.7% (9)
Genetic contamination	10.4% (8)
Resistance to herbicides or pesticides	6.5% (5)
Seed monopolies	5.2% (4)
World food trade conflicts	5.2% (4)
Impact on biological diversity	3.8% (3)
Harms to other organisms	2.6% (2)

In comparison, the risks of GMOs were mentioned much less than their advantages. With the exception of the risks of seed monopolies and world food trade conflicts, other kinds of risks associated with GMOs were strongly challenged by Chinese scientists and governors [27-29]. They argued that to date there are no established scientific evidence to prove the harm of GMOs, and that the potential risks of GMOs should be well considered, but they should not be arbitrarily quoted to impede the development and applications of GM technology.

### Attitude towards GMOs

News articles were also reviewed for their overall attitude towards GMOs, namely, whether news articles were neutral, supporting or advocating the GM technology, or negative or opposing GMOs. Results indicated that as authoritative representatives of Chinese media, articles in the *People’s Daily* and *Guangming Daily* were largely supportive of the GM technology R&D programs and GM cottons (37 articles, 48.1%), and by comparison, no articles expressed negative attitudes towards GMOs. More than half of all articles (40 articles, 51.9%) were neutral on the subject and provided descriptive news reports on GMOs.

## Discussion

This analysis contributes new findings by considering the attitude of Chinese media, specifically that of two major Chinese newspapers, towards the development and cultivation of GM technology. According to news reports in our data set, the Chinese government has been attempting to increase development of GM technology and also to establish a sound regulatory framework for GMOs, especially in regards to bio-safety management. This ambition is partly based on the realities of the Chinese context and the advantages of GMOs. As a developing country with a huge population, limited arable land, and environmental challenges, Chinese scientists have been motivated to develop new technologies, such as GM biotechnology, to help secure the national food supply and improve the quality of life for people in China. In addition, this ambition is also inspired by the pressures of an existing monopoly over the global seed market [30]. By developing GMOs with independent intellectual property rights, China hopes to break Western seed monopolies. According to newspaper reports, China has made great progress with some GMOs, e.g., GM cotton, rice and poplar [31].

It has been suggested that, in China, traditional news media plays a role in educating the public about new technologies and alleviating doubts about these innovations [15]. As such, the experts have been given this key role in regards to the safety issue of GM food. Eight newspaper articles are interview records with experts in GMO fields. These news articles include explanations of GMOs, their benefits, and the current scientific safety management and regulation of GMOs in China in a language that is easy for lay audiences to understand. Importantly, news articles also discussed that, to date, there is no scientific evidence for health problems associated with GMOs, and that the approved GM crops can be consumed without anxiety [26-29,32,33].

A considerable number of news articles seem to have been written for the purposes of educating the public about GM technology. These include descriptions regarding the technology itself, as well at the pros and cons of GM technology. It is uncertain the degree of influence that these “educational” news articles have, but if results from public opinion polls in China are any indication, it appears that Chinese consumers are still relatively unfamiliar with GM technology [32,34].

## Conclusion

Our results suggest that the Chinese print media is largely supportive of GMOs. They also demonstrate that the print media portray the Chinese government as actively pursuing national GMO research and development and the promotion of GM cotton usage. Media reports included descriptions of a legal framework for GMOs in China, including a bio-safety management system for agricultural GMOs. However, discussion of the risks or concerns associated with GMOs is minimal in the news reports. These results are in contrast to studies of news coverage of GMOs in other countries, such as the UK, USA, or Japan, where news coverage of GM technology often includes vibrant debate and depicts a variety of people and organizations in opposition to industry and, in some cases, the government [35-37].

As previously discussed, news media is a key source of health and science information for public audiences. However, there are many factors that help shape news reports of new scientific developments. Scientists and government officials also contribute to how messages are framed for public audiences, especially when the content of media reports, as seen in our results, relies heavily on expert opinions and interviews. While it is uncertain the degree to which the media in China influences public perceptions and policy development, the media, scientists, and the government are complicit actors in educating the public about new technologies, such as GMOs. As such, they should work together to ensure that science communication is accurate and balanced.

There are a number of limitations to our study. First, we only examined articles from 2 major Communist newspapers in China. Second, we only searched the titles for keywords. Third, the influence of media coverage on laypersons was out of scope of the study. Further research is required to understand how a broader sample of Chinese media (e.g., local newspapers, Internet, television, etc.) portrays GMOs and the role of the media in shaping both public opinion and policy development in a Chinese context.

## Competing interests

The authors declare that they have no competing interests.

## Authors’ contributions

LD and CR designed the study, LD collected and analyzed data, and both authors contributed to and approved the final version of the manuscript.

## Pre-publication history

The pre-publication history for this paper can be accessed here:

http://www.biomedcentral.com/1471-2458/12/326/prepub
